# Animals on Screen: Representations and Anthropomorphism in Australian Preschool Television

**DOI:** 10.3390/ani16111706

**Published:** 2026-06-02

**Authors:** Kaye Ahern, Bradley P. Smith

**Affiliations:** School of Health, Medical and Applied Sciences, College of Psychology, Central Queensland University, Adelaide Campus, 44 Greenhill Road, Wayville, SA 5034, Australia; kayeahern@gmail.com

**Keywords:** anthropomorphism, animal representation, children’s television, preschool children, content analysis, media portrayals, human–animal relationships

## Abstract

Animals feature regularly on children’s television, exposing children to implicit and explicit messages about the natural world. Although animal representation and anthropomorphism have been examined across children’s media, film, literature, and popular culture, less is known about how animals are portrayed in preschool television, particularly in relation to biological and ecological accuracy. This study examined animal representations in programs aired on ABC Kids, the Australian Broadcasting Corporation’s free-to-air children’s television channel, using quantitative and qualitative content analysis. A total of 947 episodes from 92 programs were analyzed, with a subset of 265 episodes (43 h and 45 min) from 39 programs examined in greater detail. Animals were prominently featured, appearing on screen for 88% (171 h and 40 min) of total programming time, with programs featuring animals as central characters accounting for 41% (81 h). Nearly all central animal characters exhibited anthropomorphic traits, such as walking, talking, dressing, or behaving like humans. Inaccuracies in species depiction were common, including portrayals of animals in human-like social structures, unrealistic environments, and mixed predator–prey groupings, alongside a hierarchy based on roles and relationships. These findings indicate that realistic portrayals of animals and the natural world were uncommon. The study highlights the need for greater consideration of how animal characters are represented in preschool television, and how imaginative animal portrayals might be balanced with biologically and ecologically grounded depictions. Further research should examine how such portrayals influence children’s understanding of animals and their connection with nature.

## Simple Summary

Animals appear frequently on children’s television, shaping how young viewers understand the natural world, yet little is known about how animals are portrayed in Australian preschool television or how accurately these portrayals reflect real animals and their environments. This study examined animal representations across all Australian free-to-air television programs for pre-school-aged children airing at the time of analysis, covering 92 programs and nearly 1000 episodes. Animals appeared for most of the total viewing time, with many programs featuring them as central characters. However, these animals were almost always depicted in human-like ways, including talking, wearing clothes, and living in social groups that do not occur in nature. Inaccuracies were common, such as unrealistic environments and unlikely combinations of predator and prey species. The findings highlight the need for greater consideration of how animal depictions in children’s television balance imaginative storytelling with representations of real animals and their environments, and how such portrayals may influence children’s understanding of the living world and their connection with nature.

## 1. Introduction

Non-human animals (hereafter, ‘animals’) play a significant and influential role in the lives of humans [1]. The human–animal relationship begins in infancy and manifests itself in a variety of forms throughout a human’s development [2]. More than 60% of Australian households own a pet, and among these, up to 82% are families with children [3]. As such, intimate interactions between children and animals occur from birth through activities such as play, caretaking, and co-sleeping. Animals are also prominently featured on children’s clothing, toys, art and media and observed in the wild, on farms and in zoos [2]. Perhaps one of the most prolific ways children are exposed to animals, regardless of personal ownership, is through children’s media [4].

Storytelling is an essential part of human nature and child development, exposing children to diverse perspectives and ideas and helping shape their worldview [5]. Animals have long occupied a central place in storytelling traditions that later shaped children’s literature. From Aesop’s fables to folktales and children’s fiction, talking animals have historically been used to entertain, instruct, moralize, and explore human social life through non-human characters [6,7]. Over the years, storytelling has progressed from drawings in caves to oral tales conveyed by cultural leaders, elders, parents, and teachers to written tales via books and, more recently, mass media, such as television [8]. Since its first Australian broadcast in 1956 [9], television has been one of the most widely consumed media by children [10,11].

Television ownership is almost universal in households, with fewer than 1% of families with children aged four to five years reporting that they do not have one [12]. With the rise of subscription services such as iView, Disney+, and Netflix, television content is even more readily accessible via portable devices such as laptops, tablets and mobile phones. The amount of time children spend watching television (a ‘screen-time’ activity) is at historical highs. In fact, excessive screen time is reported by Australian adults as one of Australia’s top 10 child health problems [13] with children, aged four to five years, spending more than the national recommendation of two hours per day (an average of 132 min) on screen-time activities each day [12].

Television can influence children in both negative and positive ways. Media-effects and social learning frameworks suggest that children may learn from observed models, particularly when content is repeated, engaging, and developmentally salient [14,15]. However, media effects are not uniform and vary according to content, context, child characteristics, and outcome; some areas of media-effects research, including links between violent media and aggression, remain contested [16]. In the present study, this literature is used only to support the broader premise that television can provide symbolic models and social information, rather than to imply direct or causal effects of animal portrayals on children’s behaviour. High levels of television and screen time have also been associated with developmental outcomes in young children, including language, memory, and academic skills [17].

A range of studies have examined educational and prosocial television content (e.g., kindness and sharing) and its influence on children [18,19,20]. Mares and Woodard [19] concluded that prosocial television can encourage greater tolerance and helpfulness, reduce aggression, and promote positive social interactions. Further supporting this, Coyne et al. [15] found that exposure to prosocial media content was associated with greater positive behaviour, including prosocial behaviour and empathic concern, and lower levels of negative behaviour, such as aggression. Television has frequently been associated with academic learning, such as reading and mathematics [11]. For instance, increased language skills have been observed in children aged over two years who watched *Sesame Street* [21]. More recent work extends these findings beyond prosocial and academic outcomes, demonstrating that children’s television can also model adaptive coping and resilience. For example, Bohl et al. [22] found that resilience is frequently embedded in narrative interactions and modelled through character relationships in programs such as *Bluey*, particularly within family contexts.

Television exposes children to a greater range of models in a symbolic environment, and these models convey considerable information about values, thinking, and behaviour [14]. In children’s television programs, characters are often depicted as animals [23], thus animals serve as models of behaviour for children. There are many facets to consider regarding what children are being exposed to and learning from these animal models. Television viewing in pre-school-aged children is also influenced by how the child identifies with the character and their ability to distinguish real from pretend behaviour and content [10]. Pre-school-aged children reportedly have difficulties when transferring information from educational media and applying it to real-world experiences [24], especially if iconicity (the similarity of the image portrayed and the real world) is low [24,25]. Picture-book research further demonstrates that media portrayals can shape children’s early conceptual understanding of animals, with Waxman et al. [26] finding that picture-book reading influenced how urban children construed the relationship between humans and non-human animals.

Most media (namely books and television) that children are exposed to feature animals [23,27]. A study of over 1000 children’s storybooks found that 48.5% had an animal as a central character [27]. At an even higher rate, 87% of British children’s television programs featured animals, of which 62% played a major role [23]. Many reasons may explain the prominent use of animals, including a general preference for stories with animal characters [28], given children’s innate affinity for animals [4]. Importantly, animal characters allow stories to be told in an ageless, raceless, and classless way [23], potentially making them more relatable to children. The use of animals in media can also provide space for reflection when the story is uncomfortable or distressing [29]. For instance, Smit and Smith [30] show that animal characters can help children engage with complex and emotionally challenging topics, such as death and dying, by creating psychological distance while maintaining emotional relevance.

A common approach when featuring animals in media is to portray them with human-like characteristics, a concept known as anthropomorphism [29,31]. Urquiza-Haas and Kotrschal [32] define anthropomorphism as the “attribution of human characteristics or behaviour to any other nonhuman entity in the environment” (p. 167). In the popular program *Peppa Pig*, anthropomorphism is pervasive, with animal characters displaying human physical traits (e.g., upright locomotion), behaviors (e.g., speech, driving, employment), and social roles (e.g., family structures, schooling, and community participation). These portrayals position animals as functional equivalents of humans within everyday social environments.

Anthropomorphism also has a long history in animal storytelling, from Aesop’s fables to folklore, children’s literature, and screen media, where animal characters are used to entertain, teach moral lessons, and make complex social ideas accessible to children [4,7,23,27,29]. In this sense, anthropomorphism should not be treated as inherently misleading or undesirable. Indeed, in animal behaviour and cognitive ethology, carefully applied anthropomorphic reasoning has been defended as a useful way of generating testable hypotheses about animal minds and behaviour, while avoiding the opposite error of anthropodenial, or the refusal to recognize meaningful continuities between humans and other animals [33,34]. Recent anthrozoological work similarly distinguishes anthropomorphism from anthropocentrism, emphasizing that human-like interpretation is not necessarily the same as reducing animals to human purposes or values [35]. Moreover, attributing human-like capacities to animals may support ethical recognition by emphasizing similarity, continuity, and shared emotional capacities between humans and other animals [4,32,36,37]. However, anthropomorphism becomes more complicated when fictional or symbolic animal portrayals are children’s primary exposure to particular species, especially when those portrayals replace or obscure biologically and ecologically accurate information [5,31,38]. The issue is therefore not whether animal characters should ever be anthropomorphized, but how such portrayals are balanced with opportunities to understand animals as animals.

There is limited research examining the use of anthropomorphism in children’s television [11], and existing findings are mixed. Some studies suggest that anthropomorphic representations can facilitate learning. For example, Geerdts [31] argued that more constrained forms of anthropomorphism, such as anthropomorphic language without highly unrealistic visual depictions, may support children’s biological learning and help foster perceived connections between humans and the natural world. Similarly, Geerdts et al. [28] reported that children were able to acquire factual knowledge about real animals even when information was presented in an unrealistic, anthropomorphized format. Despite these insights, little is known about how anthropomorphism is represented in children’s television content, particularly in widely accessible formats such as free-to-air programming. Understanding the nature and extent of these portrayals is therefore critical for evaluating their potential influence on children’s learning and perceptions of animals.

Children have a natural tendency to be attracted to animals [28,32], and anthropomorphic portrayals are often used to enhance the relatability and accessibility of stories. For example, Marriott [27] found that animals in children’s storybooks are frequently domesticated, centered on children’s daily experiences, and transformed in ways that make narratives more familiar and engaging. However, evidence regarding the educational value of anthropomorphism is mixed. Larsen et al. [5] found that pre-school-aged children showed greater prosocial behaviors after hearing a story with human characters compared to one featuring anthropomorphized animal characters, suggesting that anthropomorphized characters may not facilitate the transfer of lessons to real-world contexts as effectively as human representations. Similarly, Ganea et al. [38] argue that anthropomorphism can lead to misconceptions about animals and promote a more human-centered understanding of the natural world. Notably, however, these effects appear to depend on the degree of anthropomorphism, with more extreme or unrealistic portrayals being associated with reduced learning. Consistent with this, Conrad et al. [39] found that anthropomorphic language did not uniformly impair children’s learning about unfamiliar animals, further suggesting that the effects of anthropomorphism depend on context, form, and degree. More moderate forms may therefore support engagement without necessarily compromising understanding, highlighting the importance of examining how anthropomorphism is represented in children’s media.

Paul’s [23] study of children’s television in Britain found that more than 50% of animals were featured in a human-like way. More recently, Bonus [24] explored children’s science television programs, testing the portrayal of insects in educational television. He found that educational material was rarely portrayed realistically, with most programs using anthropomorphic information. Mares and Acosta [20] also noted that 92% of the pro-tolerance children’s programs included in their review featured anthropomorphic animal characters such as dogs, cats, or dragons. These studies suggest that as children explore the natural world through media, they are regularly exposed to animal depictions that vary in their biological and ecological accuracy. Recent analyses show that environmental content is relatively limited in both frequency and airtime, is predominantly conveyed implicitly through dialogue and imagery, and is often not fact-based [40].

Documenting these portrayals matters because children’s television is not merely entertainment, but a recurring source of symbolic models, social meanings, and information about the world. Existing research suggests that children can learn from media portrayals, but that learning and transfer are shaped by the degree of realism, iconicity, and similarity between media content and the real world [14,24,25]. In relation to animals specifically, anthropomorphic portrayals may support engagement, empathy, and perceived connection with animals, but may also contribute to misconceptions when fictional human-like traits obscure species-specific behaviour, ecological needs, or habitat relationships [5,28,37,38]. Establishing what children are exposed to is therefore an essential first step in determining which representations may be educationally useful, which may be misleading, and which warrant further experimental investigation.

More broadly, scholars have examined animal representation and anthropomorphism across film, animation, literature, mass media, and popular culture, including the influence of anthropomorphized animals on attitudes toward wildlife and the cultural construction of nature in Disney and other screen media [41,42,43]. This literature demonstrates that animal portrayals in media are not neutral, but can shape how audiences understand, value, and relate to animals. However, comparatively less attention has been given to systematic analysis of animal representation in preschool television, particularly within Australian free-to-air programming and with specific attention to biological and ecological accuracy.

The consequences of anthropomorphism are therefore likely to be mixed. While human-like portrayals may foster engagement, empathy, and moral concern for animals, they may also encourage inaccurate assumptions about animals’ ecological needs, behaviour, or intentions when applied uncritically to real-world animals [32,37].

Like storybooks, children’s television featuring animals can influence children’s relationships with animals and the natural world. The prominence and portrayal of animals on television have the potential to influence what children learn about and their attitudes towards animals. Sprafkin et al. [44], for instance, showed that children who watched an episode of *Lassie* with pro-social themes were more likely to help an animal they believed to be distressed. Similarly, Eagles and Muffitt [45] found that children who viewed wildlife programs reported higher attitude scores related to kindness and compassion towards wildlife.

Animal depictions can influence children’s early knowledge acquisition, as well as their interest in and engagement with animals. As attitudes develop, both children and adults tend to prefer animals perceived as more human-like in their behavioral, cognitive, or physical characteristics (e.g., primates, dogs, and dolphins), as well as those considered ‘cute’ or useful [46]. These preferences are reinforced through anthropomorphized portrayals, particularly when animals are depicted in child-like or endearing ways [46]. Importantly, attitudes and behaviors toward animals formed in childhood often persist into adulthood [29,45], with implications for areas such as animal welfare and conservation, where greater resources are frequently allocated to species perceived as appealing or useful [47]. In addition, children’s relationships with animals have been linked to aspects of wellbeing [46]. Together, these findings highlight the importance of understanding how animals are portrayed and the messages children receive through television.

Despite the prevalence of animals in children’s media, systematic research examining how animals are depicted in preschool television remains limited, particularly within Australian free-to-air programming and with attention to biological and ecological accuracy. While some insights can be drawn from children’s literature, film, and broader media studies, these are of limited applicability given that television is a dynamic, multimodal medium, and preschool programming is designed for a distinctive developmental audience. Given children’s strong connection with animals, the ubiquity of animal characters in media, and the influential role of television in early development, further investigation in this area is warranted. In this sense, children’s television provides an important form of mediated or virtual contact with animals, through which children may develop ideas about what animals are like, how humans relate to them, and how animals should be valued within society.

This study addresses this gap by adopting an exploratory approach to examine how animals are represented in Australian free-to-air television programs designed for children aged two to six years. Using qualitative content analysis, it explores key themes including anthropomorphism, animal–human relationships, behaviors, and environmental context. In doing so, it provides insight into the nature and extent of animal portrayals, children’s exposure to these representations, and how such portrayals may inform or potentially misinform children’s developing understandings of animals and the natural world.

## 2. Materials and Methods

### 2.1. Data Source

The data used in this study included children’s television programs aired on the Australian Broadcasting Corporation (ABC) free-to-air channel, ‘ABC Kids’. ABC Kids airs programs for children ages two to six years daily from 5 a.m. to 7.30 p.m., totaling 14.5 h per day [48]. All content aired during this window was recorded for 14 consecutive days, commencing 5 a.m. on 24 June 2021 and concluding 7.30 p.m. on 7 July 2021, resulting in a total recorded broadcast window of 203 h (see Table A1 and Supplementary File S1 for a complete list of programs). Within this recording period, 947 identifiable program episodes from 92 programs were included in the analysis, representing 195 h and 48 min of coded program content. The difference between the total recorded broadcast window and the coded program duration reflects non-episode material occurring between programs, such as station announcements, promotional segments, transitions, and other short interstitial content that was not included in the analysis. Episodes were electronically stored on a hard drive via the researcher’s smart television, as well as an external hard drive for the duration of the study.

### 2.2. Research Design and Analysis

To explore the representation of animals in television programs, a quantitative content analysis and a qualitative media analysis were conducted over two parts. Part 1 involved examining the programs and episodes based on program and episode descriptions, and Part 2 involved viewing episodes and conducting an ethnographic analysis. These methods are explained in turn.

### 2.3. Part 1: Quantitative Analysis—Prominence of Animals on Television

Programming information for the programs included in this study was obtained from the ABC programming guide (www.iview.abc.net.au), website (www.abc.net.au/abckids), and individual program websites. All available scheduling and program information that appeared during the recording period from these sources was manually entered into a Microsoft Excel file. This included the program title, synopsis, and imagery, as well as episode title, synopsis, imagery, duration, date, and time aired.

#### 2.3.1. Programs Featuring Animals

To identify whether animals were featured, the program title, synopsis, and imagery from the individual program websites were examined. It was determined whether an animal was mentioned in the program title or synopsis (yes/no), by name (e.g., *Bluey*, *Peppa Pig*), species (e.g., dog, cat, dinosaur), or in broader terms (animal, pet), or whether an animal appeared in the program imagery (yes/no). For programs that did not reference animals at this stage, the episode information, including the episode title, synopsis and imagery for all episodes recorded, was reviewed. *Baby Jake*, for example, is a program that does not reference an animal in program information. However, in the episode information, a monkey, a penguin and a rabbit were mentioned.

#### 2.3.2. Program Type, Style and Purpose

Programs were coded by program type (fiction, nonfiction), style (animation, live action, puppetry), and purpose (educational, entertainment). For definitions of these categories, refer to Table A2. The program synopsis and imagery were used to determine these categories for each program.

Program purpose was coded according to the dominant or explicit purpose of the program. We acknowledge that entertainment and education are not mutually exclusive categories. Many children’s programs that are primarily designed for entertainment also convey moral, social, emotional, or educational messages. For coding consistency, programs were classified as educational where explicit instruction, factual learning, or skill development was central to the program structure, and as entertainment where any learning opportunities were implicit, incidental, or embedded within a primarily fictional narrative.

#### 2.3.3. Animal Character Role

The program title and synopsis were used to identify whether each program featured an animal, human, or other creature in a central role. Central characters were defined as those occupying either a lead or support role within the narrative. Lead characters were identified as the primary protagonists and were typically featured in every episode. Support characters were defined as secondary but prominent figures (e.g., best friend, sidekick, or companion to the protagonist) who played a consistent and meaningful role across episodes. Refer to Table A3.

Where the role of an animal character was unclear, additional information was obtained from official program websites and general internet sources. For example, in *Bluey*, Bluey was coded as the lead character, whereas in *Kazoops!*, the pig Jimmy Jones was coded as a support character to the human protagonist.

#### 2.3.4. Program Information (Descriptive Details)

Program classifications, series number, episode number, duration, air date and time were collected and used as identifying information throughout the analysis (see Supplementary File S1). These details were used to identify duplications over the two-week duration. No episodes were duplicated over the two-week duration. A general website search was conducted for programs where any of the above information was not identifiable so that a full data set could be collected and analyzed.

### 2.4. Part 2: Ethnographic Content Analysis—The Representation of Animals on Television

Out of the 14-days of recording, 7 consecutive days (24 to 30 June 2021) of recorded children’s television programming were viewed and analyzed to identify themes in relation to animal representation. Such information could not be gained from details presented in the program or episode information, which was used in Part 1. Only episodes from programs where an animal was a central character (identified in Part 1) were viewed and analyzed as part of the ethnographic content analysis (see Table A4 for a list of programs viewed in Part 2). A total of 265 episodes from 39 programs, totaling 43 h and 45 min of content, were viewed and analyzed. Given that each program aired a different number of episodes per day across weekdays and weekends, there were slight differences in the number of episodes included per program. The number of episodes for each program averaged 7 episodes, ranging from 1 to 14. The duration of each program’s episodes also varied, ranging from 3 to 27 min in length.

An ethnographic content analysis approach outlined by Altheide and Schneider [49] was used to make detailed observational notes specific to the central animal character for each episode. This included developing a coding protocol (see Appendix C and Supplementary File S1) to use when making observational notes on the central characters’ relationships with other characters, their roles and identifiable traits, experiences and behaviour, the habitats in which they lived, and anthropomorphic features. The coding protocol was developed by viewing a random sample of 30 program episodes and making observational notes about how the central animal character was depicted. These initial observations were reviewed and grouped into categories to allow for a degree of consistency in the notes made for each episode by K.A. The coding protocol was then reviewed by B.S, refined by consensus, and then finalized. Following this, all episodes marked for inclusion in the ethnographic analysis were viewed and coded, guided by the coding protocol.

Data from the observations were then analyzed following the data analysis process outlined by Altheide and Schneider [49]. The observational notes for each episode were reviewed, compared and used to summarize the notes for each episode so that there was one holistic summary per program. Following this, similarities, differences, and any extremes were distinguished at a category level and category summaries were noted. Comparisons were then made within the category summaries to identify trends and patterns.

## 3. Results

### 3.1. Part 1: Prominence of Animals on Television

Out of the 92 programs examined in Part 1 (see Appendix A), 82% (*n* = 75) featured animals, 13% (*n* = 12) had no animals, and the remaining 5% (*n* = 5) featured animal-like characters (such as animal-shaped creatures, or humans dressed as animals). From the 75 programs that featured animals, 77% (*n* = 58) featured animals in every episode over the two weeks, and the remaining 23% (*n* = 17) featured animals in some episodes (23%, 74/317 episodes examined) (Table 1).

The coded program content represented 195 h and 48 min over the 14-day recording period. Programs that featured animals in every episode made up 70% (136 h 56 min) of coded program time. When combined with programs that featured animals in some episodes, animals were viewable on screen for 88% (171 h 40 min) of total daily programming time. Episodes where an animal was depicted as a central character accounted for 41% (41 h 24 min) of the total daily programming schedule (Table 1).

Across all programs (*n* = 92), the majority were fictional (*n* = 82; 89%), for entertainment (*n* = 64; 70%) and animated (*n* = 54; 59%). Out of the 58 programs that featured animals in every episode, 95% (*n* = 55) were classified as fictional, for entertainment (*n* = 43, 74%) and animated (*n* = 39, 67%). In contrast, programs with no animals (*n* = 12) were mostly fictional (*n* = 8, 67%), entertainment (*n* = 7, 58%), and live action (*n* = 6, 50%) (Table 1).

Animals were featured as a central character in half of all programs (46/92). This equates to 79% (46/58) of programs that featured an animal in all/every episode, 61% (46/75) of programs that featured an animal in all/every or some episodes (Table 1).

A variety of species appeared as lead and support characters. Across all programs (*n* = 92) humans appeared as a lead character in 44% (*n* = 41 programs), compared to animals that appeared as a lead character in 34% (*n* = 31). However, animals appeared more frequently (*n* = 26, 28%) as support characters compared to humans (*n* = 13, 14%). Out of the programs that depicted an animal as a lead character (*n* = 31 programs), wild animals were portrayed as leads in the majority of programs (*n* = 19, 61%), followed by companion animals (*n* = 5, 16%) (Table 2).

The most common animal species for lead roles were rabbits (*n* = 6, 19%), followed by dogs (*n* = 5, 16%). Out of the programs that depicted an animal as a support character (*n* = 26 programs), wild animals were portrayed as leads in most programs (*n* = 13, 50%), again followed by companion animals (*n* = 8, 31%). In these programs, the most common animal species were dogs (*n* = 6, 23%), followed by rodents (*n* = 5, 19%). Among the programs that depicted a human lead character (*n* = 41), animals were often cast as supporting characters (*n* = 12, 29%). Of these programs (*n* = 12), 42% (*n* = 5) depicted the animal species as a wild animal, 33% (*n* = 4) as a companion animal (dogs or cats), and 25% (*n* = 3) as a farm animal. When considering the programs that depicted an animal lead character (*n* = 31), humans were less frequently cast as supporting characters (*n* = 4, 13%), whereas animals more frequently supported lead animal characters (*n* = 11, 35%). See Table 2.

### 3.2. Part 2: The Representation of Animals on Television

Program descriptions were examined to provide an overview of the frequency with which animals appeared. This identified 46 programs that featured animals in a central role. The aim of Part 2 was to explore in greater detail the roles and depictions of the identified central animal characters, which could not be achieved in Part 1. As such, one week’s worth of programming (*n* = 39 programs; see Table A4) was viewed and analyzed using an ethnographic content analysis (see Appendix C). When viewing the television episodes, observations were made about the central animal character in relation to anthropomorphic features, character relationships, and the setting.

#### 3.2.1. Anthropomorphism

Across all programs, nearly all central animal characters (37/39, 95%) were anthropomorphized in some way. The degree to which anthropomorphism was employed varied greatly (see Table 3 for examples and Table 4 for a breakdown across all programs). In 41% of programs (*n* = 16), the central animal character was depicted with predominantly human-centric features, or put another way, a very limited presentation of species-specific traits. In these programs, the central animal character walked on two legs (bipedally) to mimic human gait rather than the gait naturalistic to their species (which was generally quadrupedal); talked and communicated like a human, performed human-like actions such as sitting at a dining table to eat food, lying in a bed to go to sleep, or going to school; and dressed in human-like clothes or wore/used accessories such as a hat or bag. A further 54% (*n* = 21 programs) depicted the central animal character with a mix of human- and species-like features. To give an example, *Peter Rabbit*, depicted the central character—a rabbit named Peter, exhibiting prey-like characteristics typical of its species (e.g., hopping, escaping natural predators such as foxes), yet this character also wore clothes, walked bipedally, spoke like a human and behaved in a human-like way (e.g., jumping on a bed). In another program, *Peppa Pig*, the central animal character, a pig named Peppa, made species- like sounds (snorting), but in all other ways acted, dressed and spoke as a human.

Dogs were the only species depicted with few to no human-like traits, and just 5% (*n* = 2) depicted the central animal character in this way. In these programs (*Noddy Toyland Detective*, *The Most Magnificent Thing*), the dog characters supported a human lead character as a companion animal, and/or had pet-like qualities (e.g., subservient to a human). These characters could not speak, instead making species-like sounds (barking, growling), were quadrupedal, and did not wear clothes. No trends were identified based on program type (fiction, non-fiction), purpose (educational, entertainment) or style (animated, live action, puppetry)—anthropomorphic elements were observed across each of these categories.

Central animal characters were often depicted as able to think, feel, reason, and see another’s point of view. For example, 85% of all programs viewed (*n* = 33/39) depicted a central animal character who demonstrated, through body language but mostly by vocalizing, feelings that were similar to those experienced by humans such as happiness, sadness, envy, disappointment, excitement, and anger/frustration. The plots of these episodes (and sometimes programs) tended to center around the characters learning to experience their feelings. For example, in an episode of *Daniel Tiger’s Neighbourhood*, Daniel Tiger felt overwhelmed by noisy and rough play and learnt that when feeling overwhelmed, finding a quiet place to calm down helps. In the remaining programs (*n* = 6, 15%), the central animal characters showed limited human-like thinking, feeling, and reasoning. These characters were cast alongside a human, usually as a pet, and the human character was depicted to have thinking, feeling, and reasoning capabilities. For example, in an episode of *Kazoops!* Jimmy Jones (pig) comforted Monty (his human owner) when Monty was upset about his inability to whistle. Jimmy Jones’ role was as a supportive presence, listening to Monty vocalize his feelings of disappointment and sadness.

Central animal characters were depicted with a wide variety of human-centric traits as opposed to species-like traits such as predator or prey-like natures. Many (*n* = 28, 72%) were observed to be curious or inquisitive, playful, thoughtful, helpful, and friendly as they engaged in childlike activities such as going to school or playing with friends. For example, *Peppa Pig* depicted a pig that was playful, friendly, curious about the world, and showed child-like honesty in her comments to friends and family. In *The Adventures of Paddington*, Paddington Bear was sweet-natured, thoughtful of others, a keen helper, affectionate, clumsy, and curious about the human world. The depiction of these traits along with the experiences in which they were demonstrated (e.g., a visit to the beach, or in search of a football in a neighbor’s yard) was considered to better reflect a human child than an animal. Few programs showed animals with human-like and species-like natures (*n* = 5, 13%). A notable example of a blended nature was *Peter Rabbit*, which depicted a rabbit as brave, a leader, clever, resourceful, and thoughtful, who escaped natural predators (prey-like) and stole food from a human’s vegetable patch (pest-like). In 15% (*n* = 6 programs), animals demonstrated few to no human-centric traits depicted as either inanimate or support characters.

At times, central animal characters were depicted in employment or performed a role that benefited others (*n* = 6, 15%). This included a rat that owned a shop (*Bananas in Pyjamas*); a cat that worked at a wildlife sanctuary (*Andy’s Wild Adventures*); native Australian animals that were recruits with the surf rescue lifesaver service (*Kangaroo Beach*); a mouse that was a king’s knight (*Sir Mouse*); a polar bear sea captain that rescued sea animals (*Octonauts*); and quokkas that helped explore animal facts (*The Wonder Gang*).

Central animal characters also engaged with non-central animal characters in employment. The most featured non-central character species that held jobs included dogs (*n* = 3 programs, 8%), rabbits (*n* = 3, 8%), and mice (*n* = 3, 8%). For example, in *Peppa Pig*, a rabbit bus driver, shop assistant, and community center coordinator was depicted. In *Becca’s Bunch*, Becca was served by a rabbit in a supermarket. In *Noddy Toyland Detective* and *Becca’s Bunch*, mice (Clockwork Mouse and Mayor Lady Mouse, respectively) were the town mayors. Dogs were also depicted in roles such as sea captain (*Lily’s Driftwood Bay*), doctor, veterinary nurse, and shop assistant (*Bluey*).

Many central animal characters were gendered (*n* = 35, 90%). In half of all programs the characters were male (*n* = 20, 51%), 21% were female (*n* = 8), and 23% (*n* = 9) had dual central characters that were a mix of male and female (e.g., Bop and Boo in *Brave Bunnies*) and 5% (*n* = 2) had an unknown gender.

#### 3.2.2. Relationships Between Animals and Other Characters

Many central animal characters (*n* = 27, 69%) were observed in unnatural friendship groups consisting of multiple species of animals that would not cohabitate in real life. For example, in *Brave Bunnies*, rabbits formed friendships with tigers, kangaroos, llamas, crocodiles, giraffes and peacocks. In 44% (*n* = 17), friendship groups consisted of prey and predatory animals. For example, The *Adventures of Paddington* depicted a bear and pigeon as friends and *Becca’s Bunch* depicted a bird, worm, squirrel and fox as friends. Further to this, 15% (*n* = 6) depicted a school or learning setting where a variety of animal species were grouped together. For example, in *Hey Duggee*, a rhinoceros, crocodile, octopus, hippopotamus, and mouse were taught by an adult dog.

Featuring animals in family structures familiar to children was common. In 26% of programs (*n* = 10), central animal characters lived with their mum and/or dad, and siblings of the same species. For example, in *Peppa Pig*, Peppa Pig’s family consisted of Mummy Pig, Daddy Pig, her brother, George, Grandpa and Grandma Pig. Just one family was blended with the central animal character adopted (*Dinosaur Train*). A further 10% (*n* = 4) depicted the central animal characters with a human family. Of these, two (*The Adventures of Paddington, Kazoops!*) depicted the central animal character in a family structure consisting of a human mum, dad, son, and daughter. The other two programs featured a pet dog with a mum and daughter family structure, and a pet seagull with a dad and daughter structure. Only one of these four programs (*The Adventures of Paddington*) featured the animal as the lead character; the remaining three featured a human as the lead.

Central animal characters often had endearing and positive traits such as friendliness, kindness, and helpfulness. In all programs (*n* = 39, 100%) the central animal characters were liked by other characters, evident in their language (e.g., positive and inclusive language), facial expressions (e.g., smiling and open expressions) and body language (e.g., affectionate). At times, some animals, specifically rodents and monkeys, highlighted wayward characteristics. Examples include Rat in *Bananas in Pyjamas*, who often tricked other characters to benefit himself, and in *Peter Rabbit*, a non-central rat character tricked another out of their home. Sir Mouse in *Sir Mouse* had a temper that went off with a bang; her character at times exploded from anger. Non-central monkeys were labelled as ‘naughty’ in *Curious George* and *Hey Duggee*. Foxes were depicted as cunning and showed predatory natures, for example *Peter Rabbit* overcame a fox who tried to catch him for his dinner, and a fox in *Patchwork Pals* wanted to ‘gobble up’ the other characters. Central animal characters were also observed rescuing prey animals from predatory fish and birds. For example, in *Octonauts* the central animal character rescued prey animals from predatory seagulls and fish.

In many instances (17/39, 44%), animals displayed ownership over other animals. This consisted of central animal characters who were pets (*n* = 5, 13%), or owned a pet (*n* = 4, 10%), and a further 21% (*n* = 8) featured a non-central animal that was a pet. Central animal characters that were viewed as pets included dogs (*Noddy Toyland Detective, The Most Magnificent Thing*), a pig (*Kazoops!*), a seagull (*Lily’s Driftwood Bay*), and sheep (*Shaun the Sheep*). These animals were subservient or owned by a human character and were non-verbal. The central animal characters that owned a pet animal included a dog with a pet cat (*Hey Duggee*), a rabbit with a pet dog (*Miffy’s Adventures Big and Small*), and a tiger and pig with pet fish (*Daniel Tiger’s Neighbourhood, Peppa Pig*). The pets exhibited species-like behaviour and appeared in realistic habitats (e.g., the pet fish were non-verbal, did not wear clothes, and swam inside a fishbowl). A variety of species were observed as pets to non-central animal characters including birds (*Peppa Pig, Noddy Toyland Detective*); invertebrates (*The Hive, Peter Rabbit*); a horse (*Miffy’s Adventures Big and Small*); dogs and cats (*Shaun the Sheep, Pins and Nettie*); fish and lizards (*Bluey*). All these animals were subservient to an animal owner, except for the dog and cat in *Shaun the Sheep*, which were subservient to a human owner. Only the pets in *Shaun the Sheep* demonstrated human-like behaviour such as holding and drinking from a cup.

The concept of death was broached in a few programs (*n* = 3, 8%), and the deceased animal species included a fish (*Daniel Tiger’s Neighbourhood*), bird (*Bluey*), and rabbit (*Peter Rabbit*). These programs depicted the central animal character experiencing and processing emotions such as sadness/grief. For example, in *Daniel Tiger’s Neighbourhood*, upon noticing the deceased pet fish, the lead character was encouraged by his parents to ask questions, and draw a picture of his deceased pet. While in *Bluey*, the central animal character found a wild, injured bird that subsequently died at a veterinary clinic. To process her feelings, the lead character asked questions, hugged her parents and used pretend play to replay the situation. In *Peter Rabbit*, the central animal character often reminisced about his deceased father and spoke of his father’s things (e.g., tunnels, bed); he also used his father’s journal and tunnels.

#### 3.2.3. Animal Environments and Habitats

Only a few programs (*n* = 2, 5%) depicted an environment that was a natural habitat to the central animal species. More often (*n* = 37, 95%), the programs featured animals in human-centric settings such as schools, playgrounds, or houses; or in settings that had human-centric elements such as kitchens, train stations, and front doors; or fantastical/made-up settings such as a cloud-land where flowers floated, or a toy-land with animated toys. Table 5 presents examples of some of the differing types of settings depicted.

## 4. Discussion

The present study examined how animals are depicted in Australian free-to-air television programs for preschool-aged children and found that animals are highly prevalent and systematically portrayed in anthropomorphic and ecologically unrealistic ways. Animals featured extensively across programming, appearing in the majority of airtime and frequently occupying central narrative roles, regardless of program type, purpose, or style. Although research examining animal representation in children’s television is limited, particularly within an Australian context, the available evidence suggests similar patterns across media. For example, Paul [23] reported that animals appeared in 87% of British children’s television programs, with 62% featuring animals as central characters, closely comparable to those observed in the present study (82% and 61%, respectively). Similar patterns have also been identified in children’s storybooks [27]. Taken together, these findings suggest that the prominence of animals in children’s media is both widespread and enduring, reflecting a consistent reliance on animal characters as central narrative devices across time and contexts.

A broad range of animal species was depicted, with dogs, rabbits, and mice most commonly represented as central characters. This pattern is consistent with children’s storybooks [27]. In television contexts, Lerner and Kalof [50] similarly identified dogs as one of the most depicted animals in advertising, although rabbits and mice were less prominent. The frequent use of dogs may reflect their familiarity and cultural significance, particularly within Australia, where they are the most common household pet [51]. Dogs are often perceived as relatively similar to humans in their social behaviour and cognition [46], enhancing their suitability as relatable and engaging characters. Similarly, rabbits may be favored due to their perceived ‘cuteness’ and resemblance to human infant features (e.g., large eyes, rounded facial features) [52,53], which are known to elicit positive emotional responses and increase engagement.

From a media production perspective, the selection of familiar and appealing species likely reflects both developmental considerations, such as enhancing relatability and engagement, and commercial priorities that favor easily recognizable and marketable characters. However, this familiarity may come at a cost. The repeated emphasis on a narrow range of familiar and appealing species may limit children’s exposure to the diversity of the animal world and reinforce anthropocentric or selective representations of species. This is particularly relevant in a context where children are spending increasing amounts of time engaging with screen-based media while spending less time interacting with nature directly [53,54]. As a result, mediated representations may play a growing role in shaping children’s exposure to and understanding of different animal species.

The repeated use of species and associated traits may contribute to simplified representations of animals. Animals were often depicted with consistent and reduced characteristics, such as foxes being portrayed as cunning or predatory, while other species, such as rabbits, were frequently depicted as positive and endearing protagonists. These patterns are important because they show that children’s television does not represent animal species neutrally, but often attaches particular narrative roles, values, or traits to specific species. For example, rabbits are widely recognized as an invasive pest species in Australia [55], yet in the programs examined, they were commonly portrayed in favorable and child-friendly ways. These findings highlight the importance of considering not only the frequency with which animals are represented, but also how specific species are selected and characterized. Future research should examine whether repeated exposure to such portrayals influences children’s perceptions of different species, including native, domestic, and invasive animals.

The ethnographic analysis (Part 2) provided further insight into how animals were depicted, highlighting considerable variability in representational styles across programs. Despite this variation, three consistent areas of depiction emerged—anthropomorphism, relationships, and environmental context—which collectively illustrate how animals are constructed within children’s television. These patterns extend the quantitative findings by demonstrating not only the prevalence of animals but also the ways in which they are systematically portrayed in human-like and ecologically unrealistic ways.

### 4.1. Anthropomorphism

Anthropomorphism emerged as a defining feature of animal representation across programs, with most central animal characters depicted as human-like to some degree. This finding is consistent with prior research across children’s media, including both television and storybooks [4,20,23,27,50,52]. Central animal characters were typically named and portrayed as walking, talking, dressing, and expressing emotions in ways that closely resembled humans. They were often situated within familiar, child-oriented contexts and engaged in everyday activities characteristic of human childhood.

In some cases, anthropomorphism was so pronounced that animal characters functioned as near-equivalents of human children. For example, in *Bing*, the central character could be replaced with a human child with little change to behaviour, environment, or narrative structure. The use of animal characters in this way may enhance relatability, as animals can operate outside of social categories such as race, class, and age [23]. Anthropomorphism has also been suggested to play a role in children’s developing sense of self, enabling exploration of both human and non-human perspectives [4].

Notably, invertebrates were represented as central characters in several programs and were depicted with pronounced human-like traits. This contrasts with earlier findings by Paul [23], where invertebrates were typically confined to minor or more animal-like roles. The increased prominence and humanisation of these species may reflect a shift in representational practices, potentially driven by novelty or creative differentiation. Given that insects are often associated with negative emotions such as fear or disgust, these portrayals may contribute to more positive perceptions by making them more relatable and socially engaging [37,56].

In addition, most central animal characters were explicitly gendered. This finding is consistent with broader research in children’s media, where male characters are often overrepresented and treated as the default [4,50]. However, it contrasts with findings from some studies of children’s storybooks, where gender is often less clearly defined [27]. The assignment of gender to anthropomorphized animals reflects broader social norms, as gender functions as a fundamental organizing principle in human societies [50]. Together, these findings suggest that gendered patterns observed in other forms of children’s media persist within television contexts.

### 4.2. Relationships Between Animals and Other Characters

Central animal characters were typically depicted within social structures that closely resembled human relationships, including nuclear families (e.g., parents and siblings) and friendship groups. The use of these human-centric structures resulted in animals being positioned within unlikely and biologically unrealistic groupings, such as multi-species friendships and the absence of predator–prey dynamics. While such portrayals contribute to inaccurate representations of animal behaviour and ecology, they also provide a framework through which human social values, such as friendship, cooperation, and family relationships, are modelled. In this way, animal characters may function as vehicles for social learning, offering children opportunities to observe prosocial behaviours in line with social learning theory [14,15,50]. These relational structures further reinforce anthropomorphism, as animals are not only depicted as behaving like humans, but as participating in human-like social systems.

Across programs, central animal characters were almost universally portrayed positively within their relationships, consistently depicted as friendly, likeable, and socially accepted. This contrasts with earlier findings by Paul [23], where portrayals varied more distinctly by species, with companion animals more frequently positioned as ‘good’ characters than wild or farm animals. One implication of these uniformly positive depictions is that they may reinforce simplified or idealised views of animals, particularly for children whose direct experiences are largely limited to familiar domestic species, especially companion animals [50]. While positive portrayals may support empathy and prosocial attitudes toward animals and potentially benefit conservation efforts [47], they may also contribute to misunderstandings about animal behaviour. For example, consistently depicting animals as approachable and non-threatening may lead to the underestimation of risk, increased anthropomorphic projection of human emotions and intentions [32], and the inappropriate application of these assumptions in real-world interactions [37,52].

A further pattern was the emergence of an implicit ‘hierarchy of animals’, in which different species were systematically assigned particular roles or narrative functions. Animals from certain phylogenetic groups (e.g., invertebrates, reptiles, fish, and some mammals such as foxes) were more likely to be depicted as subordinate, disposable, or instrumental to the storyline—for example, as pets, as targets of predation, or in narratives involving danger or death. Similar patterns have been identified in earlier research, where wild animals, particularly reptiles and invertebrates, were more frequently cast as antagonistic or less valued characters [23]. These recurring representational hierarchies may reinforce broader human value systems, in which animals perceived as less similar to humans are assigned lower moral status [52]. Given that human preferences tend to favour animals that are perceived as cute or cognitively and behaviourally similar to humans [46], such portrayals may have downstream implications for conservation priorities, where more appealing species receive disproportionate attention and resources [47].

### 4.3. Animal Environments and Habitats

Animals were frequently depicted in environments that were ecologically unrealistic or removed from their natural habitats. This occurred in several ways. First, consistent with prior research [4], animals from diverse species and geographic regions (e.g., sheep, zebras, dogs, pandas, and mice) were commonly shown co-existing within the same setting, despite having incompatible natural habitats. Second, animals were often placed in imagined, fantastical, or child-oriented environments, such as schools, playgrounds, or domestic spaces, or situated entirely within human homes and neighbourhoods, reflecting patterns also observed in children’s storybooks [27]. Even when elements of a natural habitat were present, these environments were frequently modified with human-centric features, such as furnishings or domestic objects (e.g., a rabbit’s burrow equipped with a kitchen).

The limited depiction of animals within accurate ecological contexts suggests that children are frequently exposed to representations of animals that are disconnected from the natural world. This is particularly relevant given broader societal trends indicating that children are spending less time engaging directly with nature and more time interacting with screen-based media [54]. Although many of the programs examined are designed primarily for entertainment rather than formal education, repeated media portrayals may still contribute to the background ideas children develop about animals, their habitats, and their relationships with humans.

Developing a connection with nature has been associated with a range of positive outcomes for children, including benefits for mental health and wellbeing, social and physical development, and pro-environmental attitudes [46]. The frequent portrayal of animals in decontextualized or human-centric environments may therefore shape how children understand animals and their relationship to the natural world, particularly when such portrayals are not balanced by exposure to animals in more realistic ecological contexts. However, this does not mean that imaginative depictions necessarily displace realistic representations, or that all domestic settings are inaccurate; companion and farm animals do live in human-associated environments. Rather, the concern is that repeated portrayals of animals outside plausible biological or ecological contexts may provide children with fewer opportunities to encounter animals as species with distinct habitats, behaviours, and environmental relationships.

### 4.4. Implications of Anthropomorphising Animals on Children’s Television

The practical significance of these findings lies in the scale and consistency of children’s exposure to animal portrayals. Because animals appeared across most of the sampled programming and were frequently central to the narrative, these depictions may provide repeated models through which children encounter animals, animal behavior, and human–animal relationships. The present study cannot determine whether these portrayals have positive or negative effects on children. However, existing evidence suggests several plausible pathways of influence, including effects on factual learning, transfer of knowledge to real animals, empathy and moral concern, species preferences, perceived risk, and connection with nature.

Across the ethnographic analysis, anthropomorphism emerged as a central and pervasive feature of animal representation in children’s television. Consistent with prior research, anthropomorphic portrayals were widespread and varied in degree, shaping how animal characters were constructed and understood [28,38]. Importantly, anthropomorphism is not inherently problematic. Its effects are likely to depend not only on the degree of anthropomorphism but also on its form, context, and narrative purpose. For example, relatively constrained forms, such as anthropomorphic language used alongside otherwise realistic animal information, may not necessarily interfere with children’s learning about animals and may help foster perceived connections between humans, animals, and the natural world [28,31].

A growing body of research suggests that early human–animal connections play an important role in children’s development, influencing wellbeing and shaping long-term attitudes toward animals [29,45,46]. In this context, anthropomorphic animal characters can be effective vehicles for communicating prosocial messages and modelling social and emotional experiences relevant to childhood. Their familiarity and appeal make them particularly engaging, enabling children to connect with narratives centred on friendship, family, cooperation, and care [4,28]. As such, animals remain a central and meaningful component of children’s media environments, even when depicted in human-like ways.

However, the findings of the present study suggest that anthropomorphism is often applied in ways that may undermine children’s understanding of real animals and the natural world. Highly anthropomorphised depictions frequently present animals as behaving, communicating, and existing in ways that are biologically inaccurate. Anderson and Henderson [57] similarly argue that children’s attachment to fictional animals may contribute to distorted expectations of real animals’ intelligence, capabilities, morality, and behaviour. Given that children learn through observing and modelling behaviour [14,15], these portrayals may limit the extent to which children can transfer knowledge from media to real-world contexts. Some empirical evidence supports this concern, indicating that highly anthropomorphic representations can be associated with reduced learning about animals [5,38], particularly when iconicity is low and representations deviate substantially from reality [24,25]. At the same time, this relationship is not straightforward. Geerdts [31] concluded that anthropomorphism may not have as strong a negative effect on children’s animal learning as previously suggested, while other research indicates that children can learn factual information from anthropomorphic animal media under some conditions [28,39].

The practical significance of this issue is illustrated by the *Peppa Pig* episode ‘Mister Skinny Legs’ (Season 1, Episode 47), which was removed from Australian broadcast and online availability after concerns that it reassured children that spiders were harmless [58]. Although this message may seem mild within a British-produced children’s program, it was considered inappropriate for Australian audiences because some Australian spiders are medically significant. This example shows how friendly or prosocial portrayals of animals can become problematic when disconnected from local biological and ecological realities. It also demonstrates that concerns about inaccurate animal depictions have already influenced broadcast decisions involving a program included in the present analysis.

Beyond learning outcomes, anthropomorphic portrayals may also reinforce human-centred perspectives of animals when they encourage children to interpret animals primarily through human social roles, needs, or values. As argued by Timmerman and Ostertag [4], attributing human characteristics to animals can implicitly position humans as the reference point against which animals are understood. At the same time, such portrayals may also reflect narrative and developmental strategies rather than simple claims about animal inferiority. Characters such as Peppa Pig, Paddington Bear, and Kip are often depicted in child-like ways: learning from adults, making mistakes, needing guidance, and navigating social situations familiar to young viewers. This may encourage children to identify with animal characters and use them as models for social and emotional learning [14,15,23]. However, because these characters’ lives are largely organised around human social norms, repeated portrayals of animals as child-like, dependent, or embedded in human-centred worlds may still encourage children to understand animals primarily in relation to human experiences and values [4].

It is important to clarify that advocating for more accurate and ecologically grounded representations does not mean that children’s animal media should abandon fantasy, humour, or anthropomorphism, nor that all animal characters should be portrayed with complete biological realism. Many children’s programs rely on imaginative animal characters to engage young viewers, model social and emotional experiences, and support storytelling. Rather, the concern is that when highly anthropomorphized portrayals dominate children’s media environments, and when they are rarely balanced by more naturalistic depictions, children may have fewer opportunities to encounter animals as animals: beings with species-specific bodies, behaviors, needs, habitats, and ecological relationships. Greater accuracy could therefore be incorporated through selective and developmentally appropriate elements, such as showing animals moving in species-typical ways, living in plausible environments, expressing species-relevant behaviors, or being contextualized within real habitats and ecological relationships, without eliminating the imaginative qualities that make children’s media engaging.

It is also important to recognize that children’s media environments are diverse, and that biologically and ecologically accurate animal content is widely available through documentaries, educational programming, nature-focused books, digital platforms, and classroom resources, including programs produced by organisations such as National Geographic. The issue is therefore not simply whether children are exposed to anthropomorphic or inaccurate portrayals, but how these portrayals are balanced with more realistic representations of animals and their environments. Parents, caregivers, and teachers may play an important mediating role in this process by helping children interpret fictional animal portrayals, distinguish fantasy from biological reality, and connect media content with real-world animal knowledge. In this sense, responsibility for children’s understanding of animals is shared across content creators, broadcasters, educators, and families.

Taken together, these findings highlight a tension inherent in anthropomorphic representation. While anthropomorphism can enhance engagement and support the communication of important social and emotional messages, its pervasive and often exaggerated use may contribute to simplified or distorted understandings of animals and their environments. Given the prominence of animals in children’s television, there is a need for greater consideration of how these portrayals balance entertainment, fantasy, and ecological or biological accuracy. Incorporating more naturalistic elements alongside engaging narratives, or providing children with a wider mix of realistic and fantastical animal portrayals, may offer richer and more accurate representations of the animal world.

### 4.5. Limitations and Future Research

Several limitations should be considered when interpreting these findings. First, the analysis was restricted to free-to-air television programs aired on ABC Kids during the selected recording period, and therefore is not representative of all children’s television content. Although multiple episodes from a large number of programs were included, it is possible that the full range of animal depictions, relationships, and settings was not captured within the study timeframe. In addition, the ethnographic nature of the analysis means that findings are shaped by the interpretations of the researcher. While efforts were made to apply a systematic coding protocol, the potential for subjective bias remains, particularly given the researcher’s prior familiarity with the programs.

Given the exploratory nature of this research, several avenues for future investigation emerge. The current study focused on free-to-air programming; however, children’s media consumption is increasingly shaped by on-demand viewing and subscription-based streaming services. Platforms such as Netflix are widely used in Australian households [59], allowing children and caregivers to selectively access content rather than relying on scheduled programming. Future research could therefore expand the sampling frame to include streaming services or focus on programs with high viewership, providing a more accurate representation of the content children are most likely to encounter. Additionally, examining differences in animal representation across age-targeted programming may offer further insight into how content varies developmentally.

Further research is also needed to examine the impact of these portrayals on children’s knowledge, attitudes, and behaviors toward animals. In particular, future studies should test whether repeated exposure to highly anthropomorphized animal characters affects children’s factual understanding of species, their ability to distinguish fictional from real animal behaviour, their willingness to approach or interact with animals, their preferences for familiar or appealing species, and their sense of connection with nature. Such work could also refine or develop child-appropriate measures of anthropomorphic thinking, drawing on existing adult measures such as the Individual Differences in Anthropomorphism Questionnaire (IDAQ; [36]), alongside age-appropriate assessments of animal knowledge, attitudes, and intended behaviour. Experimental or longitudinal designs could assess these outcomes by comparing varying levels of exposure to anthropomorphic and realistic animal representations. For example, studies similar to Dixon et al. [60], which examined the influence of food advertising on children’s attitudes and behaviors, could be adapted to explore the effects of animal-related media content. Such research could inform both content development and parental or caregiver guidance.

Finally, future research could investigate the perspectives and decision-making processes of those involved in creating children’s television content. Examining the motivations, experiences, and production practices of writers, producers, and broadcasters may provide insight into how and why particular types of animal representations are developed. Programs such as *Sesame Street* demonstrate how research-informed production processes can enhance educational outcomes [61]. Understanding these processes more broadly may have implications for industry practices, funding priorities, and the development of programming that balances entertainment with educational value.

## 5. Conclusions

This study demonstrates that animals are a prominent feature of Australian preschool children’s free-to-air television and are frequently depicted as central characters. These portrayals are highly anthropomorphic, with animals consistently represented as thinking, behaving, and socializing in human-like ways and often placed in human-centric or ecologically unrealistic environments. As a result, children are regularly exposed to simplified and human-centred representations of animals and the natural world. Given children’s increasing engagement with screen-based media, future research should examine how these portrayals may influence children’s understanding and perceptions of animals.

These findings highlight the importance of considering how animals are represented in children’s media and the potential implications for children’s learning, attitudes, and connection to the natural environment. Because children’s television provides a form of mediated contact with animals, these representations may also contribute to the broader social meanings attached to animals, including how they are understood, valued, and treated. Greater attention to balancing engaging storytelling with more accurate and ecologically grounded representations may support the development of more informed and meaningful understandings of the animal world and potentially more accurate and respectful relationships with animals beyond the screen.

## Figures and Tables

**Table 1:** Programming durations, program type definitions, purpose and style, and a breakdown based on animal appearance. For definitions, see Table A3.

	All Programs	Animals in Program				
		Yes: All Episodes	Yes: Some Episodes	Yes: Only Central Animal Character	No Animals	Other
Duration						
Minutes	11750	8216	2084	4865	956	494
Hours	195.83	136.93	34.73	41.40	15.93	8.23
% of all programs	100%	70%	18%	41%	8%	4%
Type						
Fictional	82 (89%)	55 (95%)	14 (82%)	44 (96%)	8 (67%)	5 (100%)
Non-Fiction	10 (11%)	3 (5%)	3 (18%)	2 (4%)	4 (33%)	0 (0%)
Total	92 (100%)	58 (100%)	17 (100%)	46 (100%)	12 (100%)	5 (100%)
Purpose						
Entertainment	64 (70%)	43 (74%)	11 (65%)	35 (76%)	7 (58%)	3 (60%)
Educational	28 (30%)	15 (26%)	6 (35%)	11 (24%)	5 (42%)	2 (40%)
Total	92 (100%)	58 (100%)	17 (100%)	46 (100%)	12 (100%)	5 (100%)
Style						
Animation	54 (59%)	39 (67%)	7 (41%)	35 (76%)	4 (33%)	4 (80%)
Live Action	19 (21%)	6 (10%)	6 (35%)	1 (2%)	6 (50%)	1 (20%)
Puppetry	0 (0%)	0	0	0	0	0
Animation, Live Action	10 (11%)	6 (10%)	2 (12%)	4 (11%)	2 (17%)	0
Puppetry, Live Action	6 (7%)	5 (8%)	1 (6%)	4 (11%)	0	0
Puppetry, Animation	2 (2%)	2 (3%)	0	2 (4%)	0	0
Animation, Puppetry, Live Action	1 (1%)	0	1 (6%)	0	0	0
Total	92 (100%)	58 (100%)	17 (100%)	46 (100%)	12 (100%)	5 (100%)

**Table 2:** Frequency of lead and support character types and animal species groups depicted in programs.

Character Type/Species Group	Number of Programs	
	Lead Character	Support Character
Wild Animals		
Rabbit/Hare	6	1
Rodent	1	5
Other mammals *	5	1
Bird	3	2
Insect	1	3
Bear	3	0
Fish	0	1
Total wild	19	13
Companion Animals		
Dog	5	6
Cat	0	2
Total companion	5	8
Other Animals		
Farm Animal	3	3
Toy animal	2	1
Dinosaur	2	1
Total other	7	5
Animal Total	31 (34%)	26 (28%)
Human Total	41 (44%)	13 (14%)
Made-up Creatures	20 (22%)	11 (12%)
Nil characters depicted in role		42 (46%)
Total Programs	92 (100%)	92 (100%)

* Note. “Other wild mammals” refers to wild mammal species that were not classified as rabbits/hares, rodents, or bears. This category included monkey, tiger, fox, hedgehog, and Australian native mammals such as kangaroo, koala, platypus, quokka, and wombat.

**Table 3:** Representative examples of anthropomorphic characteristics in central animal characters.

Television Program	Anthropomorphic Characteristics
*The Adventures of Paddington*	A bear depicted walking bipedally, wearing clothing, using human language (speech and gestures), and engaging in human-like behaviors (e.g., brushing teeth, completing household chores).
*Dog Loves Books*	A dog depicted walking bipedally, using human language (speech and gestures), engaging in human activities (e.g., reading books), and occasionally wearing clothing or accessories.
*Bluey*	A dog depicted walking bipedally, using human language (speech and gestures), and engaging in human-like behaviours (e.g., playing games, using toys, attending school), while also displaying some species-specific traits (e.g., tail wagging).
*Peppa Pig*	A pig depicted walking bipedally, wearing clothing, using human language (speech and gestures), and engaging in human-like behaviors (e.g., attending school, participating in a play), while retaining species-specific vocalizations (e.g., snorting).
*Kazoops!*	A pig depicted moving quadrupedally, producing species-specific vocalizations (e.g., snorting, squealing), and not using spoken language, but communicating through gestures and participating in human-like activities (e.g., playground play).
*Peter Rabbit*	A rabbit depicted alternating between bipedal and quadrupedal movement, wearing clothing, using human language (speech and gestures), and engaging in both human-like behaviors (e.g., jumping on a bed) and species-specific behaviors (e.g., avoiding predators).
*The Most Magnificent Thing*	A dog depicted moving quadrupedally, not wearing clothing, not using language, and primarily engaging in species-typical behaviors (e.g., chasing a squirrel, being walked by an owner).

**Table 4:** Summary of human-like and species-like anthropomorphic features observed for each program.

Program	Character	Species	Human-like				Animal-like			
			Talked	Walked	Behaved	Dressed	Talked	Walked	Behaved	Dressed
Very limited presentation of species-like traits										
*Bananas in Pyjamas*	Rat	Rat	Y	Y	Y	Y	N	N	N	N
*Bing*	Bing	Rabbit	Y	Y	Y	Y	N	N	N	N
*Becca’s Bunch*	Becca	Bird	Y	Y	Y	Y	N	N	N	N
*Book Hungry Bears*	Multiple	Mixed (bears)	Y	Y	Y	Y	N	N	N	N
*Daniel Tiger’s Neighbourhood*	Daniel	Tiger	Y	Y	Y	Y	N	N	N	N
*Dirtgirlworld*	Ken	Weevil	Y	Y	Y	Y	N	N	N	N
*Dinosaur Train*	Buddy	Trex	Y	Y	Y	Y	N	N	N	N
*Dog Loves Books*	Dog	Dog	Y	Y	Y	Y	N	N	N	N
*Miffy’s Adventures Big and Small*	Miffy	Rabbit	Y	Y	Y	Y	N	N	N	N
*Octonauts*	Captain Barnacle	Polar Bear	Y	Y	Y	Y	N	N	N	N
*Sir Mouse*	Sir Mouse	Mouse	Y	Y	Y	Y	N	N	N	N
*The Adventures of Paddington*	Paddington	Bear	Y	Y	Y	Y	N	N	N	N
*The Wonder Gang*	Multiple	Quokka	Y	Y	Y	Y	N	N	N	N
*Wanda And The Alien*	Wanda	Rabbit	Y	Y	Y	Y	N	N	N	N
*Brave Bunnies*	Multiple	Rabbit	Y	Y	Y	Y	N	N	N	N
*Rainbow Chicks*	Multiple	Chick	Y	Y	Y	Y	N	N	N	N
Human and Species-like traits presented (Y = yes, N = no, G = human gestures only)										
*Bluey*	Bluey	Dog	Y	Y	Y	Y	N	N	Y	N
*Andy’s Wild Adventures*	Kip	Cat	Y	Y	Y	Y	N	N	N	Y
*Kangaroo Beach*	Multiple	Mixed	Y	Y	Y	Y	N	Y	N	N
*Peter Rabbit*	Peter Rabbit	Rabbit	Y	Y	Y	Y	N	Y	Y	N
*The Hive*	Buzzbee	Bee	Y	Y	Y	Y	N	Y	N	N
*Peppa Pig*	Peppa	Pig	Y	Y	Y	Y	Y	N	N	N
*Curious George*	George	Monkey	G	Y	Y	Y	Y	Y	Y	N
*Hey Duggee*	Duggee	Dog	G	Y	Y	Y	Y	Y	N	N
*Lily’s Driftwood Bay*	Gull	Seagull	G	N	N	Y	Y	Y	Y	N
*Pins And Nettie*	Multiple	Hedgehog	G	N	Y	Y	Y	Y	Y	N
*Shaun The Sheep*	Shaun	sheep	G	Y	Y	Y	N	Y	Y	N
*Timmy Time*	Timmy	Sheep	G	Y	Y	Y	Y	N	N	N
*Pocoyo*	Multiple	Mixed	G	Y	Y	Y	N	N	N	N
*The Patchwork Pals*	Multiple	Mixed	G	Y	Y	N	Y	Y	Y	N
*Pablo*	Multiple	Mixed	Y	Y	Y	N	N	Y	N	N
*Digby Dragon*	Chips	Squirrel	Y	Y	Y	N	N	Y	Y	N
*Guess How Much I Love You*	Little Nutbrown Hare	Hare	Y	Y	Y	N	N	Y	Y	N
*Kiri and Lou*	Kiri	Dinosaur	Y	Y	Y	N	N	N	N	N
*Baby Jake*	Nibbles	Rabbit	N	Y	Y	N	N	Y	N	N
*Big Ted’s Big Adventure*	Big Ted	Bear	N	N	N	Y	N	N	N	N
*Kazoops!*	Jimmy Jones	Pig	N	N	Y	Y	Y	Y	Y	N
Very limited presentation of human-like traits										
*Noddy Toyland Detective*	Bumpy	Dog	N	N	N	N	Y	Y	Y	Y
*The Most Magnificent Thing*	Dog (name unknown)	Dog	N	N	N	N	Y	Y	Y	Y

**Table 5:** Representative examples of environmental depictions in children’s television programs featuring animal characters.

Television Program	Environmental Characteristics
*Bluey*	Depicts a human-centric environment, including a suburban neighborhood and a house with human furnishings (e.g., kitchen, fridge, sink, windows). Outdoor settings include playgrounds, toy shops, and veterinary clinics.
*The Hive*	Depicts a blended environment combining species-specific and human-like elements. The home includes human furnishings (e.g., dining table), while architectural features (e.g., hexagonal doors and windows) reflect a honeycomb structure. Outdoor settings include grass, trees, and sky, alongside human elements such as playgrounds.
*Wanda and the Alien*	Depicts a blended environment, including a rabbit’s burrow within a woodland setting that is furnished in a human-like way (e.g., beds, shelves, pictures). Outdoor environments resemble a natural habitat.
*Pocoyo*	Depicts a highly abstract environment, characterized by a blank white background. Human-like objects and settings appear intermittently, depending on the episode.
*Guess How Much I Love You*	Depicts a naturalistic environment resembling a woodland habitat typical for rabbits, with minimal to no human-like elements or furnishings.
*Rainbow Chicks*	Depicts a fantastical environment composed of clouds and imaginary landscapes, with no clear natural habitat or human-centric features.

## Data Availability

The data supporting the findings of this study, including the full coding dataset, are available as Supplementary Materials associated with this article (Supplementary File S1).

## Supplementary Materials

The following supporting information can be downloaded at https://www.mdpi.com/article/10.3390/ani16111706/s1. Supplementary File S1: Raw dataset and coding.

## Appendix A

There were 947 episodes from 92 programs included in this study, as indicated in Table A1. These programs were recorded commencing from 5 a.m. on 24 June 2021 through to 7.30 p.m. on 7 July 2021.

**Table A1:** Program titles (*n* = 92) and number of episodes (*n* = 947) that aired on ABC Kids from 24 June 2021 to 7 July 2021.

Program Title	Number of Episodes
Andy’s Safari Adventures	9
Andy’s Wild Adventures	3
Baby Jake	14
Bananas in Pyjamas	28
Becca’s Bunch	11
Ben And Holly’s Little Kingdom	3
Big Ted’s Big Adventure	7
Bing	14
Bluey	28
Bob The Builder	11
Book Hungry Bears	14
Brave Bunnies	14
Charlie And Lola	14
Clangers	14
Curious George	7
Daniel Tiger’s Neighbourhood	14
Digby Dragon	14
Dino Dana	14
Dinosaur Train	14
Dirtgirlworld	10
Dog Loves Books	14
Dot.	3
Fireman Sam	15
Five Minutes More	3
Floogals	14
Get Grubby TV	4
Go Jetters	14
Guess How Much I Love You	14
Hey Duggee	14
Hoopla Doopla!	14
Hoot Hoot Go!	3
In The Night Garden	4
Justine Clarke: I Like To Sing!	1
Kangaroo Beach	11
Kazoops!	7
Kiddets	3
Kiri And Lou	14
Lily’s Driftwood Bay	7
Little Ted’s Big Adventure	7
Love Monster	3
Miffy’s Adventures Big And Small	14
Mister Maker	3
Mister Maker Around The World	7
Molly And Mack	11
Molly Of Denali	7
Moon And Me	10
Nella The Princess Knight	14
Noddy Toyland Detective	11
Numberblocks	14
Octonauts	11
Odd Squad	10
Olobob Top	18
Pablo	4
Patchwork Pals	10
Peppa Pig	28
Peter Rabbit	12
Pins And Nettie	3
PJ Masks	14
Play School	37
Play School Art Crew	7
Play School Art Time	3
Play School Show Time	2
Play School Song Time	1
Play School Story Time	4
Play School: Acknowledgement Of Country	1
Pocoyo	7
Rainbow Chicks	11
Ready, Jet, Go!	3
Ready, Steady, Wiggle!	14
Remy & Boo	14
Rusty Rivets	3
Sally & Possum	4
Sarah and Duck	3
School of Roars	11
Sesame Street	28
Shaun the Sheep	14
Sir Mouse	4
Teletubbies	14
The Adventures of Paddington	14
The Furchester Hotel	14
The Hive	14
The Most Magnificent Thing	1
The Wiggles World	14
The Wonder Gang	14
This is Scarlett And Isaiah	4
Thomas and Friends: Big World! Big Adventures!	3
Tik Tak	11
Timmy Time	11
Twirlywoos	14
Waffle the Wonder Dog	3
Wallykazam!	7
Wanda and the Alien	11
Total	947

## Appendix B

### Appendix B.1

Table A2 outlines the categories used for program type, style and purpose along with definitions and an example program for each category.

**Table A2:** Definitions and examples for program type, purpose and style.

Category	Definitions	Examples
Program Type	Fiction: Programs that are about made-up or imaginary things. For example, the program entails made-up characters, settings, locations, experiences, events, and these things are not based on facts or real people	Dinosaur Train
	Non-fiction: Programs that are based on real people, facts, locations, and events rather than those that are made-up or imagined	Play School
Program Purpose	Educational: Programs that present factual information or provide learning opportunities about people, events, animals, locations, skills, or ideas/concepts in a way that is central to the storyline and meant to inform/teach children, i.e., through demonstration such as in play school art crew or mister maker, through semi-narration dinosaur train or exploration by a central character such as in dino dana or daniel tiger. The learning opportunities can be presented by/via fictional or imaginary characters or events.	Andy’s Wild Adventures
	Entertainment: Programs that present made-up or imaginary information about people, events, animals, locations, in a way that is meant to entertain. While there may be some moral lessons or learning opportunities, these are not overt or central to the storyline; for example, in timmy time, timmy may learn to share or work with friends at some point in an episode, but this is smaller part of a larger plot that is designed to be entertaining rather than overtly educational.	Shaun the Sheep
Program Style	Animation: Programs in which all characters, settings, events, and other elements are computer or hand-drawn images or models that move, i.e., cartoons [62]	Bluey
	Live action: Programs in which all characters are settings, events and other elements are real, for example play school is hosted by real people, and the props and other elements used are also real rather than animated by computer or in some other way.	Play School Art Crew
	Puppetry: Programs that involve puppets or toys that are shaped like people, animals or creatures that are controlled and moved by a person either via strings, with a hand inside or in some other way [62].	Sesame Street

### Appendix B.2

Table A3 outlines the categories used to code animal character roles and an example program for each category.

**Table A3:** Definitions and examples for character roles. Images from ABC iView, by Australian Broadcasting Corporation (ABC), 2021, (https://iview.abc.net.au/). All images are copyright 2021 by the ABC.

Role Definition	Example	Image Example
Lead: Characters in lead roles were considered to be the protagonist or the main central character. They are featured in every episode.	Program: Bluey Character: Bluey Program Description: Bluey is about a Blue Heeler dog that lives with her family. Bluey is the protagonist and while there are other key roles in the program such as Bingo, Bandit, and Chilli, episodes depict Bluey’s adventures and experiences.	Program: Bluey Episode date: 27 June 2021 Episode title: Postman
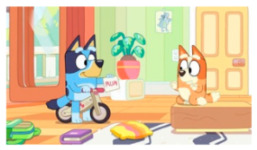
Support: Characters in support roles were classified as holding a key role; however, they were not as central as the lead character. They were often portrayed as a best friend, side-kick, or in some way help the protagonist. They tend to be featured in every episode.	Program: Kazoops! Character: Jimmy Jones Program Description: Kazoops! is about a boy, Monty, who goes on adventures with his pet pig Jimmy Jones. Monty is the protagonist, and Jimmy Jones plays the role of best friend and pet. Jimmy Jones is featured in almost every episode.	Program: Kazoops! Episode date: 26 June 2021 Episode title: Tasty Trip
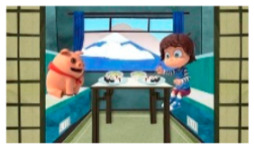
Recurring: Characters in recurring roles were classified as those that appear in more than one episode and are featured in a role that is less central to the lead and support roles. They do not appear in every episode.	Program: Bananas in Pyjamas Character: Pedro Program Description: Bananas in Pyjamas is about two Bananas who live in a town with their friends. Each episode is about the two Bananas and their adventures. In some episodes, the Banana’s adventures may involve their animal friends, for example Pedro, the pig.	Program: Bananas in Pyjamas Episode date: 1 July 2021 Episode title: The Pie
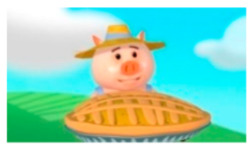
Extra/Background: Characters in extra/background roles were classified as those that appeared in an episode but not as a lead, support or recurring character. They usually have no name, may appear in the background, may be part of lead or support character’s experience / adventure in an episode, or are featured in a minor way such as a craft item that is being made.	Program: Play School Character: Puppy (unnamed) Program Description: Play School is about human hosts who demonstrate, recreate, and imagine stories, activities and plays. In some parts of an episode the hosts may be joined by an animal, for example a puppy, play with animal toys, read books about animals or make animal craft items.	Program: Play School Episode date: 2 July 2021 Episode title: Five Senses: 5
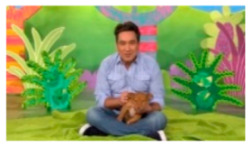

## Appendix C

### Appendix C.1

Programs included in Part 2.

**Table A4:** Program titles and frequencies of programs that featured animals in central role.

Program Title	Number of Programs	Number of Episode	Sum of Episode Duration (Minutes)
*Andy’s Wild Adventures*	1	3	44
*Baby Jake*	1	7	94
*Bananas in Pyjamas*	1	14	173
*Becca’s Bunch*	1	7	78
*Big Ted’s Big Adventure*	1	7	28
*Bing*	1	7	49
*Bluey*	1	14	106
*Book Hungry Bears*	1	7	77
*Brave Bunnies*	1	7	49
*Curious George*	1	5	133
*Daniel Tiger’s Neighbourhood*	1	7	84
*Digby Dragon*	1	7	77
*Dinosaur Train*	1	7	86
*Dirtgirlworld*	1	5	60
*Dog Loves Books*	1	7	55
*Guess How Much I Love You*	1	7	76
*Hey Duggee*	1	7	55
*Kangaroo Beach*	1	7	83
*Kazoops!*	1	7	55
*Kiri and Lou*	1	7	62
*Lily’s Driftwood Bay*	1	7	52
*Miffy’s Adventures Big and Small*	1	7	75
*Noddy Toyland Detective*	1	7	97
*Octonauts*	1	7	78
*Pablo*	1	2	28
*Patchwork Pals*	1	7	28
*Peppa Pig*	1	14	80
*Peter Rabbit*	1	6	72
*Pins and Nettie*	1	3	15
*Pocoyo*	1	7	50
*Rainbow Chicks*	1	7	44
*Shaun The Sheep*	1	7	50
*Sir Mouse*	1	2	32
*The Adventures of Paddington*	1	7	77
*The Hive*	1	7	49
*The Most Magnificent Thing*	1	1	22
*The Wonder Gang*	1	7	94
*Timmy Time*	1	7	84
*Wanda and the Alien*	1	7	74
Total	39	265	2625

### Appendix C.2. Ethnographic Content Analysis Coding Protocol Template

- Program Title
- Episode Title
- Character Name
- Character Species
- Character role
- Degree of Anthropomorphism—is the animal completely human-like, partly human-like, or animal-like?
  ○ Moving/walking
    ▪ Species-like
    ▪ Human-like
  ○ Communication/speaking
    ▪ Species-like
    ▪ Human-like
  ○ Appearance
    ▪ Species-like
    ▪ Human-like
  ○ Behaviour
    ▪ Species-like
    ▪ Human-like
  ○ Sentient
  ○ Nature (animal-like examples: wild, flighty, playful, prey-like, predatory, pet; human-like examples: personas, traits, roles, leader, boss, student, child)
    ▪ Species-like
    ▪ Human-like
- How is the central character depicted—Are they in an animal setting, human setting, made-up setting?
  ○ Setting
    ▪ Species-like
    ▪ Human-like
  ○ Distortions
- How is the central character depicted in their relationship with others?
  ○ Animal & human
  ○ Animal & Animal
  ○ Animal & Other
  ○ Family or friend stereotypes or hierarchies?
- Plot
  ○ Story
  ○ Theme
  ○ Lesson
- Miscellaneous
  ○ Comments
  ○ Questions
  ○ Comparison

## References

[1] Alves R.R.N., Barboza R.R.D., Alves R.R.N., Albuquerque U.P. (2018). Chapter 15—The role of animals in human culture. Ethnozoology.

[2] Serpell J. (1999). Guest editor’s introduction: Animals in children’s lives. Soc. Anim..

[3] Animal Medicines Australia (2019). Pets in Australia: A National Survey of Pets and People.

[4] Timmerman N., Ostertag J. (2011). Too many monkeys jumping in their heads: Animal lessons within young children’s media. Can. J. Environ. Educ..

[5] Larsen N.E., Lee K., Ganea P.A. (2018). Do storybooks with anthropomorphized animal characters promote prosocial behaviors in young children?. Dev. Sci..

[6] Cosslett T. (2006). Talking Animals in British Children’s Fiction, 1786–1914.

[7] Lefkowitz J.B., Campbell G.L. (2014). Aesop and animal fable. The Oxford Handbook of Animals in Classical Thought and Life.

[8] Morgan M., Nabi R.L., Oliver M.B. (2009). Cultivation analysis and media effects. The SAGE Handbook of Media Processes and Effects.

[9] Australian Broadcasting Corporation ABC’s First Television Broadcast. https://web.archive.org/web/20120501221842/https://www.abc.net.au/archives/80days/stories/2012/01/19/3413777.htm.

[10] Richert R.A., Robb M.B., Smith E.I. (2011). Media as social partners: The social nature of young children’s learning from screen media. Child Dev..

[11] Taggart J., Eisen S., Lillard A.S. (2019). The current landscape of US children’s television: Violent, prosocial, educational, and fantastical content. J. Child. Media.

[12] Yu M., Baxter J. (2015). LSAC Annual Statistical Report 2015: Chapter 5—Australian Children’s Screen Time and Participation in Extracurricular Activities.

[13] Australian Institute of Health and Welfare (2020). Australia’s Children.

[14] Bandura A. (2001). Social cognitive theory of mass communication. Media Psychol..

[15] Coyne S.M., Padilla-Walker L.M., Holmgren H.G., Davis E.J., Collier K.M., Memmott-Elison M.K., Hawkins A.J. (2018). A meta-analysis of prosocial media on prosocial behavior, aggression, and empathic concern: A multidimensional approach. Dev. Psychol..

[16] Coyne S.M., Stockdale L., Nelson D.A. (2012). Two sides to the same coin: Relational and physical aggression in the media. J. Aggress. Confl. Peace Res..

[17] Duch H., Fisher E.M., Ensari I., Harrington A. (2013). Screen time use in children under 3 years old: A systematic review of correlates. Int. J. Behav. Nutr. Phys. Act..

[18] Feng Y., Park J. (2015). Bad seed or good seed? A content analysis of the main antagonists in Walt Disney- and Studio Ghibli-animated films. J. Child. Media.

[19] Mares M.L., Woodard E. (2005). Positive effects of television on children’s social interactions: A meta-analysis. Media Psychol..

[20] Mares M.L., Acosta E.E. (2008). Be kind to three-legged dogs: Children’s literal interpretations of TV’s moral lessons. Media Psychol..

[21] Anderson D.R., Pempek T.A. (2005). Television and very young children. Am. Behav. Sci..

[22] Bohl K., Smith B.P., Bolling M. (2025). “Oh, Biscuits!” Exploring resilience in the children’s television program Bluey. Educ. Dev. Psychol..

[23] Paul E.S. (1996). The representation of animals on children’s television. Anthrozoös.

[24] Bonus J.A. (2019). The impact of pictorial realism in educational science television on US children’s learning and transfer of biological facts. J. Child. Media.

[25] Ganea P.A., Pickard M.B., DeLoache J.S. (2008). Transfer between picture books and the real world by very young children. J. Cogn. Dev..

[26] Waxman S.R., Herrmann P., Woodring J., Medin D.L. (2014). Humans (really) are animals: Picture-book reading influences 5-year-old urban children’s construal of the relation between humans and non-human animals. Front. Psychol..

[27] Marriott S. (2002). Red in tooth and claw? Images of nature in modern picture books. Child. Lit. Educ..

[28] Geerdts M.S., Van de Walle G.A., LoBue V. (2016). Learning about real animals from anthropomorphic media. Imagin. Cogn. Pers..

[29] Burke C.L., Copenhaver J.G., Carpenter M. (2004). Animals as people in children’s literature. Lang. Arts.

[30] Smit E., Smith B. (2023). Dealing with death: The role of animal-themed literature in supporting children with grief and loss. Hum.-Anim. Interact..

[31] Geerdts M.S. (2016). (Un)Real animals: Anthropomorphism and early learning about animals. Child Dev. Perspect..

[32] Urquiza-Haas E.G., Kotrschal K. (2015). The mind behind anthropomorphic thinking: Attribution of mental states to other species. Anim. Behav..

[33] Burghardt G. (2016). Mediating claims through critical anthropomorphism. Anim. Sentience.

[34] de Waal F.B.M. (1999). Anthropomorphism and anthropodenial: Consistency in our thinking about humans and other animals. Philos. Top..

[35] Lockwood R., Fine A., Mueller M., Ng Z., Beck A., Peralta J. (2023). Anthropomorphism and anthropocentrism in the Anthropocene. The Handbook on Human Animal Interactions and Anthrozoology.

[36] Waytz A., Cacioppo J., Epley N. (2010). Who sees human? The stability and importance of individual differences in anthropomorphism. Perspect. Psychol. Sci..

[37] Young A., Khalil K.A., Wharton J. (2018). Empathy for animals: A review of the existing literature. Curator.

[38] Ganea P.A., Canfield C.F., Simons-Ghafari K., Chou T. (2014). Do cavies talk? The effect of anthropomorphic picture books on children’s knowledge about animals. Front. Psychol..

[39] Conrad M., Marcovitch S., Boseovski J.J. (2021). The friendly fossa: The effect of anthropomorphic language on learning about unfamiliar animals through both storybooks and live animal experiences. J. Exp. Child Psychol..

[40] Morgan B.L., Smith B.P. (2024). Environmental representation on Australian children’s television: An analysis of conservation messages and nature portrayals. Conservation.

[41] Grasso C., Lenzi C., Speiran S., Pirrone F. (2020). Anthropomorphized nonhuman animals in mass media and their influence on human attitudes toward wildlife. Soc. Anim..

[42] Lutts R.H. (1992). The trouble with Bambi: Walt Disney’s Bambi and the American vision of nature. For. Conserv. Hist..

[43] Stanton R.R. (2021). The Disneyfication of Animals.

[44] Sprafkin J.N., Liebert R.M., Poulos R.W. (1975). Effects of a prosocial televised example on children’s helping. J. Exp. Child Psychol..

[45] Eagles P.F.J., Muffitt S. (1990). An analysis of children’s attitudes toward animals. J. Environ. Educ..

[46] Borgi M., Cirulli F. (2015). Attitudes toward animals among kindergarten children: Species preferences. Anthrozoös.

[47] Small E. (2011). The new Noah’s Ark: Beautiful and useful species only. Part 1. Biodiversity conservation issues and priorities. Biodiversity.

[48] Australian Broadcasting Corporation ABC Kids. https://www.abc.net.au/abckids.

[49] Altheide D.L., Schneider C.J. (2012). Qualitative Media Analysis.

[50] Lerner J.E., Kalof L. (1999). The animal text: Message and meaning in television advertisements. Sociol. Q..

[51] RSPCA Australia RSPCA Knowledgebase—How Many Pets Are There in Australia?. https://kb.rspca.org.au/knowledge-base/how-many-pets-are-there-in-australia/.

[52] King M.J. (1996). The audience in the wilderness: The Disney nature films. J. Pop. Film Telev..

[53] Schuttler S.G., Stevenson K., Kays R., Dunn R.R. (2019). Children’s attitudes towards animals are similar across suburban, exurban, and rural areas. PeerJ.

[54] Larson L.R., Szczytko R., Bowers E.P., Stephens L.E., Stevenson K.T., Floyd M.F. (2019). Outdoor time, screen time, and connection to nature: Troubling trends among rural youth?. Environ. Behav..

[55] Roy-Dufresne E., Lurgi M., Brown S.C., Wells K., Cooke B., Mutze G., Peacock D., Cassey P., Berman D., Brook B.W. (2019). The Australian National Rabbit Database: 50 yr of population monitoring of an invasive species. Ecology.

[56] Schlegel J., Breuer G., Rupf R. (2015). Local insects as flagship species to promote nature conservation? A survey among primary school children on their attitudes toward invertebrates. Anthrozoös.

[57] Anderson M.V., Henderson A.J.Z. (2005). Pernicious portrayals: The impact of children’s attachment to animals of fiction on animals of fact. Soc. Anim..

[58] Zhou N. (2017). *Peppa Pig* ‘Spiders Can’t Hurt You’ Episode Pulled off Air in Australia—Again. The Guardian.

[59] Cunningham S., Scarlata A. (2020). New forms of internationalisation?: The impact of Netflix in Australia. Media Int. Aust..

[60] Dixon H.G., Scully M.L., Wakefield M.A., White V.M., Crawford D.A. (2007). The effects of television advertisements for junk food versus nutritious food on children’s food attitudes and preferences. Soc. Sci. Med..

[61] Fisch S.M., Truglio R.T. (2000). G Is for Growing: Thirty Years of Research on Children and Sesame Street.

[62] Cambridge University Press Cambridge Dictionary. https://dictionary.cambridge.org/dictionary/english/fiction.

